# Comparative Effectiveness of the Core Components of Cardiac Rehabilitation on Mortality and Morbidity: A Systematic Review and Network Meta-Analysis

**DOI:** 10.3390/jcm7120514

**Published:** 2018-12-04

**Authors:** Nader N. Kabboul, George Tomlinson, Troy A. Francis, Sherry L. Grace, Gabriela Chaves, Valeria Rac, Tamara Daou-Kabboul, Joanna M. Bielecki, David A. Alter, Murray Krahn

**Affiliations:** 1 Toronto Health Economics and Technology Assessment (THETA) Collaborative, 200 Elizabeth Street, Toronto, ON M5G 2C4, Canada; kabboul@gmail.com (N.N.K.); george.tomlinson@utoronto.ca (G.T.); troy.a.francis@gmail.com (T.A.F.); valeria.rac@theta.utoronto.ca (V.R.); joanna.bielecki@theta.utoronto.ca (J.M.B.); 2 Faculty of Pharmacy, University of Toronto, 144 College St, Toronto, ON M5S 3M2, Canada; 3 Department of Medicine, University Health Network, 27 King’s College Circle, Toronto, ON M5S 1A1, Canada; david.alter@ices.on.ca; 4 Institute of Health Policy, Management and Evaluation (IHPME), University of Toronto, 4th Floor, 155 College St, Toronto, ON M5T 3M6, Canada; 5 Faculty of Medicine, University of Toronto, Medical Sciences Building, 1 King’s College Cir, Toronto, ON M5S 1A8, Canada; sgrace@yorku.ca; 6 Cardiac Rehabilitation and Secondary Prevention Program, Toronto Rehabilitation Institute, University Health Network, University of Toronto, 550 University Ave, Toronto, ON M5G 2A2, Canada; 7 School of Kinesiology and Health Science, York University, 4700 Keele St, Toronto, ON M3J 1P3, Canada; 8 Department of Physical Therapy, Federal University of Minas Gerais, Av. Pres. Antônio Carlos, 6627-Pampulha, Belo Horizonte, MG 31270-901, Brazil; gabisschaves@gmail.com; 9 Human Nutrition, Bridgeport University, 126 Park Ave, Bridgeport, CT 06604, USA; Tkabboul@outlook.com

**Keywords:** coronary heart disease, cardiac rehabilitation, systematic review

## Abstract

A systematic review and network meta-analysis (NMA) of randomized controlled trials (RCTs) evaluating the core components of cardiac rehabilitation (CR), nutritional counseling (NC), risk factor modification (RFM), psychosocial management (PM), patient education (PE), and exercise training (ET)) was undertaken. Published RCTs were identified from database inception dates to April 2017, and risk of bias assessed using Cochrane’s tool. Endpoints included mortality (all-cause and cardiovascular (CV)) and morbidity (fatal and non-fatal myocardial infarction (MI), coronary artery bypass surgery (CABG), percutaneous coronary intervention (PCI), and hospitalization (all-cause and CV)). Meta-regression models decomposed treatment effects into the main effects of core components, and two-way or all-way interactions between them. Ultimately, 148 RCTs (50,965 participants) were included. Main effects models were best fitting for mortality (e.g., for all-cause, specifically PM (hazard ratio HR = 0.68, 95% credible interval CrI = 0.54–0.85) and ET (HR = 0.75, 95% CrI = 0.60–0.92) components effective), MI (e.g., for all-cause, specifically PM (hazard ratio HR = 0.76, 95% credible interval CrI = 0.57–0.99), ET (HR = 0.75, 95% CrI = 0.56–0.99) and PE (HR = 0.68, 95% CrI = 0.47–0.99) components effective) and hospitalization (e.g., all-cause, PM (HR = 0.76, 95% CrI = 0.58–0.96) effective). For revascularization (including CABG and PCI individually), the full interaction model was best-fitting. Given that each component, individual or in combination, was associated with mortality and/or morbidity, recommendations for comprehensive CR are warranted.

## 1. Introduction

Coronary heart disease (CHD) is one of the most prevalent health conditions globally [1], with an estimate of 422 million prevalent cases in 2015 [2]. Cardiac rehabilitation (CR) is designed to optimize secondary prevention of CHD [3,4]. Reviews have established that CR participation is associated with approximately 20% lower cardiovascular mortality and morbidity [5].

CR has evolved from an exercise-focused program, to a comprehensive, multi-component model of care to address all CHD risk factors [6]. Indeed, learned CR societies have published statements listing the so-called “core components” of CR [3,4,7,8,9,10], to promote delivery of all evidence-based secondary prevention recommendations [11]. These have been internationally agreed through the International Council of Cardiovascular Prevention and Rehabilitation [12], namely, nutritional counseling (NC), risk factor modification (RFM), psychosocial management (PM), patient education (PE), and exercise training (ET).

A recent meta-analysis demonstrated that CR programs offering more core components achieved greater reductions in all-cause mortality than those offering less [11]. However, reviews of the effectiveness of CR to date have not considered the impact of the individual components (except exercise). Expert recommendations to deliver each core component should be tested [12], in such a way that the “complexity” of CR can be considered and to ensure there is evidence to support delivery of each component [13]. Clearly, delivery of comprehensive CR requires more human and financial resources, and thus only those components with impact should be offered. Accordingly, the objective of this review was to evaluate the comparative effectiveness of the core components of CR on mortality and morbidity, considering main and multiplicative impacts.

## 2. Methods

The systematic review was undertaken with consideration of the Cochrane Handbook guidelines [14] and reported in compliance with the extension Preferred Reporting Items for Systematic Reviews and Meta-Analyses (PRISMA) statement for Network meta-analysis (NMA) [15,16]. NMA was used to test the comparative effectiveness of the 5 CR components.

### 2.1. Information Sources and Search Strategy

Studies were identified through a systematic, comprehensive search of the following databases from inception through 27 April 2017: MEDLINE (Ovid), EMBASE (Ovid), CINHAL (Ebsco), PsycINFO (Ovid), Cochrane Database of Systematic Reviews (CDSR), Cochrane Central Register of Controlled Trials (Cochrane Central); Web of Science (SCI-EXPANDED, SSCI, CPCI-S, CPCI-SSH). Reference lists of systematic reviews and meta-analyses identified through the search were screened for additional potentially-eligible trials.

The search strategy was designed, and search undertaken, by an information specialist experienced in systematic reviews (J.M.B.) following the Cochrane systematic review methodology [14]. It included controlled vocabulary (MeSH) and natural language terms in the following concept areas: myocardial ischemia, health education, psychotherapy, smoking cessation and synonyms. No date or language limits were applied. A detailed search strategy for MEDLINE (Ovid) is provided in Supplemental File 1. The final Medline strategy was translated into syntax appropriate for each database used.

### 2.2. Inclusion and Exclusion Criteria

Randomized controlled trials (RCTs) evaluating any combination of the core components of CR were eligible for inclusion. Participants were adults who had had a myocardial infarction (MI; including MI with non-obstructive coronary arteries or cardiac syndrome X), or who had undergone revascularization (coronary artery bypass grafting (CABG), percutaneous coronary intervention (PCI)), or whom had angina pectoris or coronary artery disease established by angiography.

Studies had to include at least one of the core components of CR [4], namely NC, RFM (≥2 of dyslipidemia, hypertension, obesity, diabetes, and/or smoking), PM (e.g., stress management, social support, psychotherapy), PE (may include lifestyle counseling), ET (including at least some form of aerobic exercise), or any combination thereof [4,17]. Usual Care (UC) could include standard medical care, such as evidence-based medications at the time of randomization, but participants could not be randomized to drug therapy or to surgery.

Studies also had to report mortality or morbidity outcomes, assessed after six or more months of follow-up. The co-primary outcomes were all-cause and cardiovascular (CV) mortality. Secondary pre-specified outcomes were total MI, fatal MI, non-fatal MI, total revascularization, CABG, PCI, as well as all-cause and CV hospitalization.

Studies of patients participating in CR following heart valve surgery, heart failure, heart transplants or implanted with either cardiac resynchronization therapy or implantable defibrillators solely were excluded. Studies of participants who completed a CR program prior to randomization, who were randomized participants prior to cardiovascular surgery, or evaluated the same CR components in both arms were excluded (e.g., the only difference was the setting or type of nutritional intervention), as were non-English studies.

### 2.3. Study Selection

Two investigators (N.N.K., T.A.F. or G.C.) first independently reviewed the titles and abstracts of all identified citations. Full-texts of potentially-eligible citations were then similarly considered to establish whether they met the inclusion criteria. Finally, 2 investigators also searched the reference lists of relevant reviews and included studies. Any disagreements were resolved by consensus or consultation with another author (S.L.G.) at each stage of the review.

### 2.4. Data Extraction Process and Quality Assessment

Using a standardized data abstraction sheet, two investigators (N.N.K., T.A.F. or G.C.) also independently extracted the data for each included study (i.e., components in each arm and outcomes; the former were checked by S.L.G.), and independently assessed the risk of bias using the Cochrane assessment tool [18]. Blinding was deemed complete when outcome assessors were masked. Patient blinding was not deemed to be relevant because of the nature of the interventions.

### 2.5. Data Synthesis and Analysis

Each study arm was characterized by the combination of the 5 core components of CR delivered in that arm. A Bayesian random-effects NMA model was computed, in which the differences in outcomes between arms in a study were expressed as a function of their core components—An approach developed for complex interventions [19]. The model accounted for the correlation of treatment effects in trials with more than two arms [20].

Three increasingly complex possibilities were explored for the roles of the core components of CR: (1) a main-effects model, in which the effects of the components were additive; (2) a two-way interaction model, in which effects also depended on pairwise combinations of components; and (3) a full-interaction model, in which each possible combination of the core components had a distinct effect. The best-fitting model was chosen upon consideration of the deviance information criterion (DIC), a measure of model fit that penalizes larger models. As there was variability in the length of follow-up across RCTs (6–300 months), the model linked the probability of an outcome to the predictor variables through the complementary log–log link, with the logarithm of follow-up time as an offset. In the main effects model, the effects of core component are estimated as hazard ratios (HR) for the presence of the component versus its absence.

The effect of the core CR components was estimated for each outcome using Markov chain Monte Carlo (MCMC) implemented in JAGS in R software (version 3.5.1) with the rjags (version 4–6) and R2jags (version 0.5–7) packages. The first 75,000 iterations were discarded, and all results were based on a further sample of at least 75,000 iterations. Four chains with different initial values were run in parallel to assess convergence using the Gelman-Rubin diagnostic statistics and plots. Heterogeneity and model fit were assessed using standard approaches [21,22,23]. Results are presented as posterior medians and 95% central credible intervals (95% CrI). The MCMC simulation framework also allowed for the presentation of other summaries of key clinical and policy interest, such as the probability that a particular core component is most effective for each outcome evaluated. Minimally-informative priors were used for all parameters [19]. Analyses were done in the intention-to-treat populations, with the clinical follow-up period closest to two years.

## 3. Results

**Figure 1:**
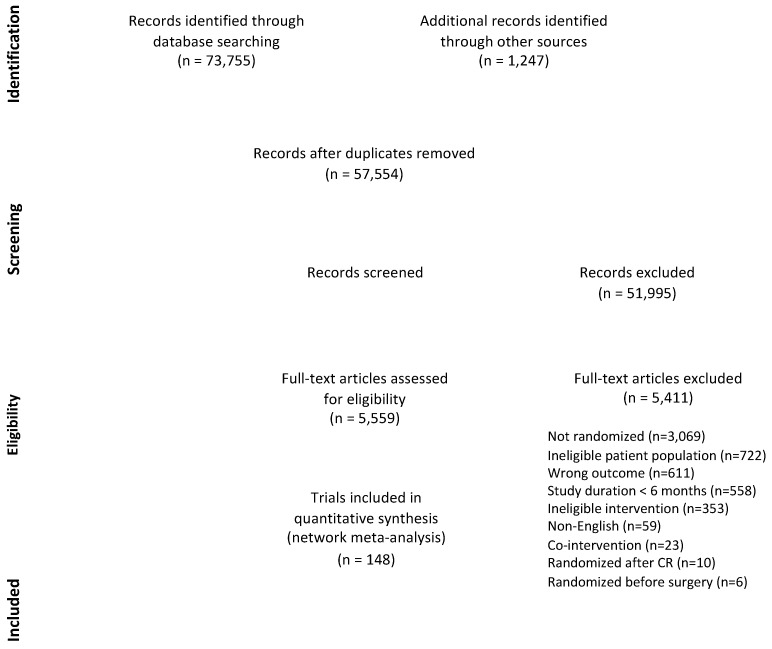
Flow Diagram.

**Figure 2:**
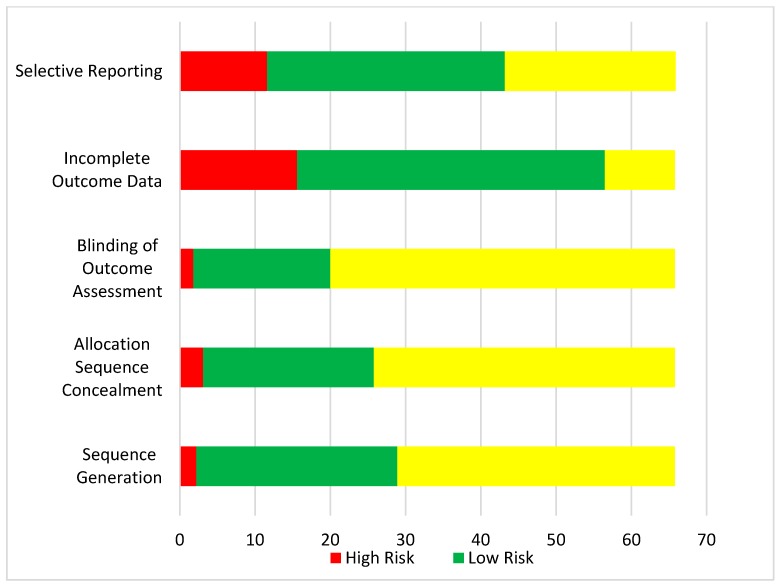
Risk of Bias in Included Trials, *N* = 148. Note: Blinding was considered low-risk when outcome assessors were masked (i.e., single-blinded). Patient blinding would not be possible given the nature of the interventions.

Figure 1 displays the process of study identification and selection. There were 148 RCTs assessing 50,965 participants included in the NMA; citations and characteristics are provided in Supplemental File 2 (References [24,25,26,27,28,29,30,31,32,33,34,35,36,37,38,39,40,41,42,43,44,45,46,47,48,49,50,51,52,53,54,55,56,57,58,59,60,61,62,63,64,65,66,67,68,69,70,71,72,73,74,75,76,77,78,79,80,81,82,83,84,85,86,87,88,89,90,91,92,93,94,95,96,97,98,99,100,101,102,103,104,105,106,107,108,109,110,111,112,113,114,115,116,117,118,119,120,121,122,123,124,125,126,127,128,129,130,131,132,133,134,135,136,137,138,139,140,141,142,143,144,145,146,147,148,149,150,151,152,153,154,155,156,157,158,159,160,161,162,163,164,165,166,167,168,169,170,171] are cited in the supplementary materials). Risk of bias assessments are shown in Figure 2. Included RCTs were undertaken between 1975 and 2017, most often in the United States (*n* = 34, 23.0%) and the United Kingdom (*n* = 16, 10.8%). Three (2.0%) were cluster RCTs [68,106,121].

Characteristics of included RCTs by outcome can be found in Table 1. Overall, 118 (79.7%) RCTs with 44,462 participants reported the primary outcome of all-cause mortality, while 42 (28.4%) RCTs with 16,770 participants reported the other primary outcome of CV mortality. The secondary endpoints were reported in 16–52 RCTs (10.8–35.1%) with 4261–16,947 participants. The mean duration of follow-up was 25.3 months (standard deviation (SD) 34.6 months).

The number of RCT arms evaluating each combination of the core components (overall and outcome-specific) can be found in Supplemental File 3. The majority of included RCTs were designed with two arms (*n* = 142, 95.9%), and six RCTs had three arms. Overall, PE was the most-frequently evaluated individual core component of CR (26 RCT arms), followed by ET (21 RCT arms). The combination of PE and RFM (12 RCT arms) was the most frequently evaluated combination of core components followed by PE and PM (9 RCT arms), the combination of PE and ET (8 RCT arms), and the combination of NC, PE, ET, PM and RMF (8 RCT arms). Usual care (no CR) was evaluated in 75 control RCT arms.

Finally, with regard to participant characteristics in included RCTs (Table 1 and Supplemental File 2), the mean age was 58.7 years (standard deviation (SD) = 6.4) and the mean proportion of males in the trials was 83.1%. Thirty-seven percent of trials (*n* = 55) included only post-MI patients.

### Effects of Core Components

Table 2 shows the model fit statistics for each model considered. For the most part, differences in the DIC between models were small, and where the difference was <2, the simpler model was preferred. The main effects model was the best-fitting model for all outcomes except revascularization (total, CABG and PCI). The 2-way interaction model had the smallest DIC for fatal MI, but as the decrease in DIC was only 2.1, the simpler main effects was considered best.

Table 3 shows the posterior medians and 95% CrIs, along with the probability that each of the CR components was the most effective, for each of the outcomes where the main-effects model was preferred. Plots of the full posterior distributions are shown in Supplemental File 4. With regard to the primary outcomes, the CR core components of PM and ET had clear benefits and were the two most effective for reducing the hazard of all-cause mortality; no core components had CrIs that excluded 1 for CV mortality (but EX, RFM and PM had HRs near 0.75 and CrIs that lay mostly below 1). With regard to secondary endpoints, the CR core components of PE, followed by ET and PM were effective for reducing the hazard of total MI, and ET for fatal MI (no effective components for non-fatal MI). The CR core component of PM was most effective for reducing the hazard of all-cause and CV-cause hospitalization.

Finally, for revascularization outcomes, the full interaction models were best-fitting; estimated HRs with respect to a control group having none of the components are shown in Table 4.

## 4. Discussion

Using methods that have not yet been applied in this field, through this review, the effectiveness of core CR components has been elucidated for the first time. Results of this NMA establish that the comprehensive delivery of the recommended core components is associated with reductions in mortality and morbidity. The core components of PM, ET and RFM each clearly reduced the hazard of mortality, with PE, ET and PM each reducing the hazard of morbidity (i.e., MI, re-hospitalization). All core components interacted synergistically to reduce revascularization.

Previous meta-analyses have demonstrated the benefits of CR in reducing mortality and morbidity [5,172,173,174,175]. However, more recent reviews have suggested that CR may have less benefit in the current era of optimal medical therapy and given advances in acute CV care [176]. The most recent update of the most rigorous of the reviews (i.e., by the Cochrane Collaboration) [5] showed CR writ large reduced CV mortality (but not all-cause), and similarly reported reductions in hospitalization. They did not report benefit for reducing revascularization, but results herein highlight the importance of offering all core components to reduce these procedures. In traditional meta-analyses, components delivered in active comparison arms are not taken into account (or the specific components in the intervention arms for that matter), which can bias towards the null. Taken together with results from the van Halwejin et al. meta-analysis showing better impact with more components [11], it can be concluded that comprehensive CR has substantial benefit in reducing mortality and morbidity.

As has been demonstrated in previous reviews [3,5,177,178,179,180,181,182], the results herein confirm the centrality of the exercise component of CR in reducing mortality and morbidity. The results also provide evidence for the first time to support other core components of CR and recommend that programs be “comprehensive”, particularly PM and PE. Previous reviews in patients with CHD of PM have only reported non-significant 7–20% reductions in all-cause and CV mortality, and have not evaluated its effects on hospitalization (all-cause or CV) [183], and of PE have reported reductions in cardiovascular events and improvements in quality of life [184]. The beneficial effects of PM observed using NMA methods were compelling.

### 4.1. Implications

Accordingly, the results of this review support guideline recommendations for the delivery of all the core components, to all indicated patients, given that the benefits have again been replicated. Given that CR is chronically under-resourced [185], many programs do not have the capacity to deliver all components however [186]. In a recent survey of all CR programs globally, PM was reported as the component least likely to be offered of those assessed herein [187]. Moreover, many programs reported limited human health resources in the area of PM (e.g., psychologists, social workers, but not nurses) [187,188,189]. Policy-makers must ensure CR is adequately resourced so not only all patients in need can access it given the additional evidence of benefits forwarded herein, but that patients receive all components, delivered by trained and regulated providers. For PM, this should likely involve depression screening, stress management and social support. Indeed, most CR guidelines call for a multidisciplinary team, comprised of healthcare professionals who have expertise covering all the core components [7].

There are some important directions for future research which flow from this work. Which combination of core components can optimize cost-effectiveness, and impacts of core components on patient-reported outcomes such as quality of life should be investigated.

### 4.2. Limitations

This review has several limitations. First, information provided in the included RCTs was often insufficient to assess their risk of bias. That patients and providers cannot be blind to arm allocation in CR RCTs cannot be overcome, however, future trials must aspire to the highest standards for conducting and reporting RCTs [190].

Second, there was some ambiguity in coding NC and PE in some trials, as they were in some cases a small part of RFM. This may have impacted the findings for these components, and hence the impact of these components on outcomes may be under-estimated. The level of detail in intervention description did seem to improve with time, but trialists are urged to report their interventions in accordance with TiDIER reporting guidelines [191].

In conclusion, using a novel approach, which takes into consideration the core components of CR, this review has reiterated the significant benefits of comprehensive CR participation in reducing mortality and morbidity. The findings herein confirm the centrality of ET as the key component of CR, and also provide strong evidence for the benefit of the other CR components, particularly PM. Policies are needed to standardize the delivery of comprehensive CR, ensuring delivery of these beneficial core components to all CHD patients.

## Figures and Tables

**Table 1:** Characteristics of included patients and trial follow-up time by endpoint.

	#	*N*	Indication (Post-MI %)	Age (Mean Years, SD)	Sex (% male)	Follow-Up (Mean Months, SD)
All Endpoints	143	69,910	55 (37.2%)	58.7 (6.4)	83.1	36.5 (38.3)
Primary Endpoints						
All-Cause Mortality	118	44,462	50 (90.9%)	58.9 (6.4)	85.6	37.2 (35.9)
CV Mortality	42	16,770	21 (38.2%)	56.9 (6.5)	92.9	47.9 (48.7)
Secondary Endpoints						
Any MI	52	16,690	28 (50.9%)	55.6 (5.4)	86.5	42.3 (43.7)
Fatal MI	16	4261	9 (16.4%)	53.7 (4.5)	100.0	47.7 (51.8)
Non-Fatal MI	31	11,919	18 (32.7%)	54.5 (4.3)	83.9	46.2 (47.3)
Any Revascularization	49	16,947	25 (45.5%)	56.8 (4.9)	83.3	34.9 (38.9)
CABG	33	7391	18 (32.7%)	56.3 (4.9)	84.4	33.0 (38.9)
PCI	23	8859	10 (18.2%)	56.8 (4.8)	87.0	27.0 (16.0)
Any Hospitalization	45	14,440	16 (29.1%)	59.0 (5.8)	82.6	31.5 (33.5)
CV Hospitalization	24	7925	10 (18.2%)	57.6 (4.1)	88.0	28.5 (30.1)

# = Number of trials reporting endpoint. *N* = number of patients randomized. SD = standard deviation. CV = cardiovascular. MI = myocardial infarction. CABG = coronary artery bypass surgery. PCI = percutaneous coronary intervention.

**Table 2:** Deviance Information Criterion by Model and Outcome.

Outcome	Model		
	Main Effects	Two-Way Interaction	Full-Interaction
All-Cause Mortality	1147.7	1147.5	1148.7
CV Mortality	415.8	415.8	417.2
Total MI	536.4	536.8	538.6
Fatal MI	143.5	141.4	142.0
Non-Fatal MI	318.4	319.3	317.9
Revascularization ^†^	545.8	545.7	537.0
CABG ^†^	319.0	319.2	314.9
PCI ^†^	239.8	237.6	236.2
All-Cause Hospitalization	549.9	549.9	551.4
CV Hospitalization	275.0	277.0	279.3

CV = cardiovascular. MI = myocardial infarction. CABG = coronary artery bypass surgery. PCI = percutaneous coronary intervention. ^†^ full interaction model better fitting. See Supplemental File 4.

**Table 3:** Estimates for Effects of Core Components and Probability of Having Largest Effect for Main Effects Model by Outcome.

Outcome	Component				
	Nutritional Counseling	Risk Factor Modification	Psychosocial Management	Patient Education	Exercise Training
All-Cause Mortality					
Estimate & 95% Credible Interval	1.07 (0.78–1.46)	0.87 (0.66–1.15)	0.68 (0.54–0.85)	0.98 (0.78–1.20)	0.74 (0.60–0.92)
Probability Best	0.01	0.04	0.67	0.01	0.28
CV Mortality					
Estimate & 95% Credible Interval	1.11 (0.68–1.74)	0.72 (0.43–1.22)	0.76 (0.53–1.11)	0.95 (0.62–1.39)	0.75 (0.53–1.05)
Probability Best	0.03	0.40	0.24	0.06	0.28
Total MI					
Estimate & 95% Credible Interval	0.94 (0.56–1.55)	0.86 (0.54–1.38)	0.76 (0.57–0.99)	0.68 (0.47–0.99)	0.75 (0.56–0.99)
Probability Best	0.08	0.10	0.17	0.45	0.20
Fatal MI					
Estimate & 95% Credible Interval	1.99 (0.57–6.86)	0.54 (0.13–2.34)	0.50 (0.21–1.13)	0.58 (0.25–1.13)	0.54 (0.31–0.87)
Probability Best	0.01	0.34	0.29	0.15	0.21
Non-Fatal MI					
Estimate & 95% Credible Interval	0.93 (0.37–2.47)	1.05 (0.37–2.68)	0.86 (0.51–1.40)	0.83 (0.42–1.47)	0.78 (0.45–1.28)
Probability Best	0.23	0.16	0.16	0.21	0.25
All-Cause Hospitalization					
Estimate & 95% Credible Interval	1.19 (0.70–1.94)	0.97 (0.67–1.38)	0.76 (0.58–0.96)	0.87 (0.63–1.18)	0.83 (0.60–1.13)
Probability Best	0.04	0.06	0.49	0.16	0.25
CV Hospitalization					
Estimate & 95% Credible Interval	0.37 (0.09–1.45)	0.70 (0.44–1.14)	0.78 (0.55–1.00)	1.03 (0.73–1.41)	0.75 (0.39–1.12)
Probability Best	0.74	0.11	0.04	0.00	0.11

CV = cardiovascular. MI = myocardial infarction.

**Table 4:** Odds Ratio Point Estimates for Core Component Combinations in Comparison to Usual Care, for Full Interaction Model for Revascularization (total).

Components	Arms (*n*)	Odds Ratio (Mean)	Odds Ratio (Median)	Credible Intervals		*p* (OR < 1)
				2.5%	97.5%	
NC	2	0.83	0.79	0.47	1.38	81.4
RFM	4	0.29	0.26	1.12	0.59	100.0
PM	5	0.93	0.93	0.66	1.24	70.2
NC, PM	2	1.26	1.11	0.40	2.96	41.5
ET	14	0.78	0.77	0.54	1.08	93.5
RFM, ET	2	1.92	1.65	0.61	4.82	16.2
PM, ET	2	0.72	0.65	0.25	1.67	83.4
NC, PM, ET	1	0.16	0.13	0.03	0.50	99.8
NC, RFM, PM, ET	1	2.49	1.76	0.36	8.89	23.4
PE	12	0.91	0.89	0.56	1.43	69.8
NC, PE	1	0.43	0.36	0.13	1.11	96.3
NC, RFM, PE	3	0.66	0.64	0.40	1.06	96.3
PM, PE	7	0.93	0.92	0.66	1.26	74.0
RFM, PM, PE	2	1.09	0.98	0.45	2.36	51.5
ET, PE	1	0.92	0.85	0.41	1.85	68.8
NC, ET, PE	2	1.38	1.07	0.29	4.03	45.9
RFM, ET, PE	1	1.44	1.29	0.48	3.26	30.9
NC, FFM, ET, PE	1	1.55	1.18	0.26	5.16	41.9
PM, ET, PE	3	0.80	0.75	0.36	1.50	78.8
NC, PM, ET, PE	1	1.07	0.80	0.20	3.45	61.8
RFM, PM, ET, PE	1	1.65	1.34	0.41	4.62	31.7
NC, RFM, PM, ET, PE	3	0.34	0.31	0.15	0.69	99.7

OR, odds ratio. NC: Nutritional Counseling; RFM: Risk Factor Modification; PM: Psychosocial Management; PE: Patient Education; ET: Exercise Training.

## Supplementary Materials

The following are available online at http://www.mdpi.com/2077-0383/7/12/514/s1, Supplemental File 1: Search Strategy for MEDLINE; Supplemental File 2: Characteristics of Included Trials; Supplemental File 3: Core Components Evaluated in Study Arms of Included Trials by Endpoint; Supplemental File 4: Graphical Display of Distribution for Each Core Component for Each Outcome (main-effects model).
