# Supplementary material for: Comparative Effectiveness of the Core Components of Cardiac Rehabilitation on Mortality and Morbidity: A Systematic Review and Network Meta-Analysis
Source: J Clin Med. 2018 Dec 4;7(12):514. doi: 10.3390/jcm7120514 (PMC6306907; doi:10.3390/jcm7120514)
Supplement: Supplementary file 1 [file jcm-07-00514-s001.pdf]

Supplemental File 1—Search Strategy in Ovid MEDLINE

1. exp myocardial ischemia/ or exp coronary artery bypass/ or ((myocard$ adj5 (ischaemia? or ischemia?)) or (isch?emi$ adj5 heart) or (myocard$ adj5 infarct$) or (heart adj5 infarct$) or angina or (coronary adj5 (disease$ or bypass or thrombo$ or angioplast$))).ti,ab.
2. exp exercise therapy/ or sports/ or physical exertion/ or exp exercise/ or rehabilitation/ or early ambulation/ or "physical education and training"/ or (rehabilitat$ or (physical$ adj5 (fit$ or train$ or therap$ or activit$)) or (train$ adj5 (strength$ or aerobic$ or exercise$)) or ((exercise$ or fitness) adj3 (treatment or intervent$ or program$)) or "cardiac rehabilitation" or kinesiotherap$).ti,ab.
3. patient education as topic/ or health education/ or psychotherapy, group/ or health promotion/ or telemedicine/ or counseling/ or self care/ or behavior therapy/ed, mt or health knowledge, attitudes, practice/ or preventive health services/ or secondary prevention/ or risk reduction behavior/ or inpatients/ed or outpatients/ed or consumer participation/ or audiovisual aids/ or "continuity of patient care"/ or ((rehabilitat$ adj5 (service$ or group$ or program$ or session$ or educat$)) or (behavio?r$ adj5 intervention$) or (health adj2 education) or (lifestyle$ adj5 (intervention$ or education$)) or "patient information" or "patient education" or "education$ intervention$" or (behavio?r$ adj5 educat$) or (educat$ adj5 rehabilitation) or ((program$ or rehabilitation or instruction$ or teach$ or learn$ or educat$) and (literature or audiovisual or "av" or internet or website$ or telecare or telemedicine or telephone or phone or teleconference or telehealth or transtelephonic$ or podcast$)) or ("public health" adj (intervention$ or program$ or scheme$)) or "education$ program$" or psychoeducation$ or educat$ or (program$ and (risk adj1 reduc$)) or (patient$ adj3 (instruct$ or teach$ or taught or learn$ or knowledge)) or "community based intervention$").ti,ab.
4. exp psychotherapy/ or exp relaxation therapy/ or exp meditation/ or exp counseling/ or stress, psychological/ or psychopathology/ or autogenic training/ or (psychotherapy$3 or (psycholog$5 adj intervent$5) or relax$6 or (counselling or counseling) or ((behavior$4 or behavior$4) adj4 (modify or modificat$4 or therap$2 or change)) or (stress adj4 management) or (cognitive adj4 therap$2) or meditate$4 or anxiety or (manage$5 adj2 (anxiety or depress$5)) or cbt or hypnotherapy$5 or (goal$2 adj3 setting) or (psycho-educat$5 or psychoeducat$5) or (motivate$5 adj3 (intervention or interv$3)) or psychopathol$4 or psychosocial$4 or distress$4 or (heart adj manual) or autogenic$5).ti,ab.
5. exp smoking cessation/ or "tobacco-use-cessation"/ or "tobacco-use-disorder"/ or tobacco-smokeless/ or exp tobacco-smoke-pollution/ or exp tobacco/ or exp nicotine/ or exp smoking/pc, th or (smoking cessation or ((quit$ or stop$ or ceas$ or giv$) adj5 smoking)).ti,ab.
6. 2 or 3 or 4 or 5
7. (randomized controlled trial or controlled clinical trial).pt. or (randomized or placebo or randomly).ab. or clinical trials as topic.sh. or trial.ti.
8. (animals not humans).sh.
9. (1 and 6 and 7) not 8

Supplemental File 2—Characteristics of Included Trials, *N* = 148

Study First Author, Year	Country	*N* Randomized	Patient Indication	Mean Age (Years)	Sex (% Male)	Control Components	Intervention Components†	Outcomes	Follow-Up (Months)
Albus, 2009 [24]	Germany	77	CAD	54.2	87.0%	RFM, PE	RFM, NC, PM, PE, ET	TM, CVM, TMI, FMI	36
Albus, 2014 [25]	Germany	570	CAD	59.0	NR	RFM	RFM, PM	TM	18
Aldana, 2007 [26]	USA	93	CAD	61.5	57.0%	RFM, PE, ET	NC, PM, PE, ET	TM	12
Allison, 2000 [27]	USA	326	Angina	58.0	56.4%	UC	RFM	TM, CVM, TMI, NFMI, REV, CABG, PCI, HOSP, CVHOSP	6
Andersson, 2010 [28]	Sweden	149	MI, CABG, PCI	53.4	NR	ET	NC, PM, PE, ET	HOSP	60
Appels, 2005 [29]	Netherlands	710	PCI	54.7	77.5%	UC	PM	TMI, REV, CABG, PCI	18
Asbury, 2011 [30]	UK	49	Cardiac Syndrome X	61.7	NR	UC	PM	TM	12
Bambauer, 2005 [31]	USA	100	MI, Angina	60.3	53.0%	UC	PM	TM	6
Barnason, 2009 [32]	USA	232	CABG	71.2	83.6%	UC	PM, ET	TM, HOSP	6
Beckie, 2010 [33]	USA	225	MI, CABG, PCI, Angina	63.5	NR	RFM, ET	RFM, PM, PE, ET	TM	6
Belardinelli_B, 2001 [34]	Italy	130	PCI	57.0	NR	UC	ET	CVM, TMI, NFMI, REV, CABG, PCI	6
Belardinelli, 2001 [35]	Italy	118	PCI	56.0	76.2%	UC	ET	HOSP	12
Bengtsson, 1983 [36]	Sweden	171	MI	56.2	43.3%	UC	PE, ET	TM, TMI	12
Beresnevaite, 2000 [37]	Lithuania	36	MI	50.1	97.2%	PE	PM	TM, CVM, TMI, FMI, NFMI, HOSP, CVHOSP	24

Berkman; 2003 [38]	USA	2481	MI	61	56,3%	UC	PM, PE	TM, CVM, NFMI, REV, CVHOSP	24
Bertie, 1992 [39]	UK	110	MI	52.4	NR	PE	NC, PE, ET	TM, TMI, REV, CABG	24
Bethell, 1999 [40]	UK	200	MI	53.7	100%	UC	ET	TM	132
Black, 1998 [41]	USA	380	MI, CABG, PCI, Angina, CAD	60.2	13.9%	NC, RFM, PE, ET	NC, RFM, PM, PE, ET	HOSP, CVHOSP	21
Blasco, 2012 [42]	Spain	203	MI, Angina	60.8	80.3%	PE	RFM, PE	TM	12
Blumenthal, 1997 [43]	USA	77	CAD	60.5	157.1%	PM, PE	ET	TM, NFMI, REV, CABG, PCI	10
Blumenthal, 2016 [44]	USA	151	CAD	61.1	94.7%	NC, PE, ET	NC, PM, PE, ET	TM, TMI, REV	63
Broadbent, 2009 [45]	New Zealand	103	MI	54.7	88.3%	UC	PM	TM,	6
Burgess, 1987 [46]	USA	180	MI	50.9	85.6%	UC	PM	TM	13
Burr, 2005 [47]	UK	3114	Angina	61.1	23.7%	UC	NC	TM, CVM	6
Burrell, 1995 [48]	Sweden	261	CABG	NR	NR	UC	PM	TM	78
Byrkjeland, 2015 [49]	Norway	137	CAD	63.9	83.9%	UC	ET	TM, CVM, HOSP, CVHOSP	12
Carson, 1982 [50]	UK	303	MI	51.5	100%	UC	ET	TM, CVM, TMI, FMI, NFMI	24
Chow, 2015 [51]	Australia	710	MI, CAD	57.6	82.0%	UC	PE	TM	6
Claesson, 2005 [52]	Sweden	198	MI, CABG, PCI, Angina, CAD	60.5	NR	UC	PM, PE	TMI, NFMI, REV, HOSP, CVHOSP	12
Clark, 2000 [53]	USA	571	CAD	71.9	NR	UC	PE	TM	12
Cohen, 2014 [54]	France	504	MI, Angina	56.8	83.9%	UC	NC, RFM, PE	TM, CVM, REV, PCI	12
Costa e Silva, 2008 [55]	Brazil	153	MI	58.3	63.4%	UC	NC, RFM, PE	TM, REV, CABG, PCI, HOSP	6

Cupples, 1999 [56]	Ireland	688	Angina	63.0	59.3%	UC	RFM, PE	TM	60
Dalal, 2007 [57]	UK	104	MI	62.4	80.8%	PM, PE, ET	NC, RFM, PM, PE, ET	TM, VER, CABG	9
Davidson, 2007 [58]	Canada	26	MI, Angina	NR	100%	PE	PM	HOSP	8
Davidson, 2010 [59]	USA	157	MI, Angina	60.2	46.5%	UC	PM	HOSP, CVHOSP	6
De Lorgeril, 1999 [60]	France	423	MI	NR	NR	UC	NC	TM, CVM, TMI, NFMI, REV	46
DeBusk, 1994 [61]	USA	585	MI	57.0	78.8%	NC, RFM	NC, RFM, PE, ET	TM, CVM, TMI, CABG, PCI	12
Donohue, 2014 [62]	USA	189	CABG	67.0	61.4%	UC	PM	TM	12
Du, 2016 [63]	China	979	MI, Angina, PCI	60.9	71.5%	PE	RFM, PE	TM, CVM, TMI, NFMI	36
Dugmore, 1999 [64]	UK	124	MI	52.2	98.4%	UC	ET	TM, NFMI	12
Edworthy, 2007 [65]	Canada	2643	CAD	63.9	72.2%	UC	RFM, PE	TM, HOSP	19
Engblom B, 1992 [66]	Finland	205	CABG	54.0	119.9%	PE	NC, PM, PE, ET	TM, CVM	12
Erdman, 1983 [67]	USA	80	MI	51.0	NR	UC	NC, RFM, PM, ET	TM, TMI, NFMI, REV, CABG, PCI	6
Fihn, 2011 [68]	USA	703	CAD	67.7	97.7%	UC	RFM, PE	TM	12
Frasure-Smith, 1985 [69]	Canada	453	MI	58.2	100%	UC	PM	TM, CVM, HOSP, CVHOSP	12
Frasure-Smith, 1987 [70]	Canada	1376	MI	59.3	65.6%	UC	PM, PE	TM, CVM, TMI, NFMI, REV	12
Fridlund, 1992 [71]	Sweden	150	MI	56.3	67.3%	UC	PM, PE, ET	TM, TMI, HOSP	12
Friedman, 1984 [72]	USA	300	MI	53.25	NR	PE	PM, PE	TM, CVM, NFMI	66

Froelicher, 1984 [73]	USA	146	CAD	53.0	100%	UC	ET	TM, TMI, REV, CABG	12
Furber, 2010 [74]	Australia	222	CAD	66.0	68.0%	PE	PE, ET	TM	10
Furze, 2012 [75]	USA	142	Angina	64.4	52.8%	RFM	RFM, PM, PE	TM, CVM, REV, HOSP, CVHOSP	6
Ghroubi, 2012 [76]	Tunisia	68	PCI, MI	NR	NR	UC	NC, RFM, PM, PE, ET	HOSP	24
Giallauria, 2008 [77]	Italy	61	MI	55.5	72.1%	UC	ET	TMI, NFMI, REV, HOSP, CVHOSP	6
Giannuzzi, 1993 [78]	Italy	95	MI	50.5	NR	PM, PE	ET	TM, REV, CABG	6
Giannuzzi, 1997 [79]	Italy	80	MI	53.5	95.0%	UC	ET	TM, CVM, REV, HOSP, CVHOSP	6
Gortner, 1988 [80]	USA	67	CABG	61.5	80.6%	PE	RFM, NC, PM, PE, ET	TM	6
Gulliksson, 2011 [81]	Sweden	362	MI, PCI, CABG	61.5	76.5%	UC	PM	TM, TMI, FMI, NFMI	96
Gutschker, 1977 [82]	Germany	70	MI	NR	100%	UC	ET	TM	24
Hamalaine, 1991 [83]	Finland	456	MI	55.8	77.0%	PE	NC, RFM, PE, ET	TM, CVM, TMI, FMI	72
Hambrecht, 1993 [84]	Germany	62	Angina	53.5	141.9%	UC	NC, ET	TM	12
Hanssen, 2007 [85]	Norway	288	MI	60.2	80.9%	UC	PE	TM	6
Harald, 2015 [86]	Netherland	754	ACS	NR	NR	UC	RFM, PE	CVHOSP	12
Haskell, 1994 [87]	USA	300	PCI, CABG, CAD	57.2	72.0%	UC	NC, RFM, PE, ET	TM, REV	48
Hawkes, 2013 [88]	Australia	430	MI	60.6	74.7%	PE	RFM, PM, PE	TM	6
Heller, 1993 [89]	Australia	450	MI	58.5	71.8%	UC	NC, RFM, PE, ET	TM	6
Higgins, 2001 [90]	Australia	99	PCI	NR	94.9%	PE	RFM, PM, PE, ET	TM	12

Hoffman-Bang, 1999 [91]	Sweden	93	PCI	53.0	78.5%	UC	NC, PM, PE, ET	HOSP	12
Jolly, 2007 [92]	UK	525	MI, CABG, PCI	61.05	76.6%	PE, ET	PM, PE, ET	TM	12
Jones, 1996 [93]	UK	2328	MI	NR	NR	UC	PM	TM, TMI, REV, PCI, HOSP	12
Kallio, 1979 [94]	Finland	375	MI	54.2	80.3%	UC	NC, RFM, PM, PE, ET	TM, CVM, NFMI	36
Karlsson, 2007 [95]	Sweden	224	MI, CABG	63.5	76.8%	RFM, PE, ET	NC, RFM, PM, PE, ET	TM	12
Koertge, 2008 [96]	Sweden	247	MI, PCI, CABG	62.0	NR	UC	PM	TM	24
Kure, 2014 [97]	Australia	602	CAD	69.6	70.3%	UC	PM	TM	30
La Rovere, 2002 [98]	Italy	95	MI	51.5	100%	UC	RFM, PE, ET	TM, TMI, NFMI, REV, CABG	120
Leemrijse, 2016 [99]	Netherlands	374	MI, Angina	60.5	81.0%	UC	RFM, PE	TM	6
Lehmann, 2011 [100]	Germany	105	CAD	59.3	70.5%	PE	NC, PM	TM, CVM, TMI, REV, CABG, PCI	36
Leizorovicz, 1991 [101] †	France	182	MI	50.0	NR	UC	NC, RFM, PM, ET	TM, TMI, CABG	24
Lewin, 1992 [102]	UK	176	MI	55.8	71.6%	UC	PE, PE, ET	HOSP	12
Lewin, 2002 [103]	UK	142	Angina	67.2	59.9%	PE	PM, PE	TM	12
Lidell, 1996 [104]	Sweden	116	MI	56.3	87.1%	RFM	NC, RFM, PM, PE, ET	TM, TMI, REV, CABG, PCI, HOSP	12
Lie, 2007 [105]	Norway	203	CAD	62.0	81.8%	PE	PM, PE	HOSP	6
Lin, 2017 [106]	Iran	288	CABG	75.5	66.3%	UC	PE	TM	18
Manchanda, 2000 [107]	India	42	CAD	51.5	100%	RFM	NC, PM, ET	REV, CABG, PCI	12
Marcus, 1999 [108]	Romania	129	MI	52.5	100%	UC	RFM, ET	TM, TMI, REV, CABG, PCI	300

Maroto-Montero, 2005 [109]	Spain	180	MI	51.4	100%	PE	PM, PE, ET	TM, CVM, TMI, FMI, REV, PCI	120
Marra, 1985 [110]	NR	167	MI	49.9	NR	PE	ET	TM, CVM, TMI, REV, CABG	54
Mayou, 2002 [111]	UK	114	MI	58.1	78.1%	UC	PM, PE	TM	12
Meisinger, 2013 [112]	Germany	340	MI	75.4	60.0%	UC	RFM, PE	TM, HOSP	12
Michalsen, 2005 [113]	Germany	101	PCI, CAD	59.4	77.2%	PE	NC, PM	REV, CABG, PCI, HOSP, CVHOSP	12
Miller, 1984 [114]	USA	198	MI	52.0	NR	UC	ET	TM, NFMI, REV	6
Mittag, 2006 [115]	Germany	343	MI, CABG, PCI	59.8	81.9%	PE, ET	NC, PM, PE, ET	TM	12
Mohiuddin, 2007 [116]	USA	209	MI, Angina	54.5	62.7%	UC	RFM, PM	TM, CVM, TMI, HOSP, CVHOSP	24
Moholdt, 2012 [117]	Norway	30	CABG	62.6	80.0%	ET	NC, PE, ET	TM, CVM	7
Molino-Lova, 2013 [118]	Italy	140	CAD, CABG	74.3	66.4%	UC	ET	TM, CVM	12
Muniz, 2010 [119]	Spain	1757	MI, Angina	62.8	76.7%	UC	PE	TM	6
Munk, 2009 [120]	Norway	40	PCI, Angina	59.0	82.5%	UC	ET	TMI, NFMI, REV, CABG, PCI, HOSP, CVHOSP	6
Murphy, 2009 [121]	Ireland	903	CAD	NR	NR	UC	RFM, PE	HOSP	18
Mutwalli, 2012 [122]	Saudi Arabia	49	CABG	56.9	100%	UC	PE, ET	TM, CVM, TMI, FMI, HOSP, CVHOSP	6

Myers, 2000 [123]	Switzerland	25	MI, CABG	55.5	100%	UC	NC, PE, ET	TM, CVM, FMI, HOSP, CVHOSP	14
Naughton, 2000 [124]	USA	641	MI	NR	NR	UC	ET	TM	36
Neubeck, 2011 [125]	NR	144	MI, Angina	64.0	74.3%	UC	RFM, PE	TM	48
Oerkild, 2012 [126]	Denmark	40	MI, CABG, PCI	76.9	57.5%	RFM	NC, RFM, ET	TM	12
Oldenburg, 1985 [127] †	Australia	46	MI	56.0	89.1%	UC	PM, PE	TMI, REV, HOSP, CVHOSP	12
Oldridge, 1991 [128]	Canada	201	MI	52.8	88.1%	UC	PM, PE, ET	TM	12
Oranta, 2010 [129]	Finland	103	MI	59.6	70.9%	UC	PM	TM	18
Ornish, 1990 [130]	USA	48	CAD	57.9	75.0%	UC	NC, PM, PE, ET	TM	12
Orth-Gomer, 2009 [131]	Sweden	239	MI, CABG, PCI	61.5	NR	UC	PM, PE	TM	108
Otterstad, 2003 [132]	Norway	197	MI, CABG, PCI, Angina	54.5	82.2%	UC	RFM, NC, PM, PE, ET	TM, HOSP	24
Park, 2013 [133]	South Korea	58	PCI, Angina, MI	58.1	82.8%	UC	RFM, PE	HOSP	6
Raffo, 1980 [134]	UK	24	Angina	50.0	100%	UC	ET	TMI	6
Rahe, 1975 [135]	USA	60	MI	49.8	88.3%	UC	PM, PE	TM, TMI, NFMI, REV, CABG, HOSP, CVHOSP	18
Rakowska, 2015 [136]	Poland	81	MI	53.5	60.5%	RFM	RFM, PM	TM, CVM, TMI, FMI, NFMI,	30
Redfern, 2009 [137]	Australia	208	MI, Angina	64.5	51.4%	UC	RFM, PE	TM	12
Rollman, 2009 [138]	USA	302	CABG	64.0	38.7%	UC	PM	TM, HOSP	8

Roman, 1983 [139]	Chile	193	MI	57.6	90.2%	UC	ET	TM, CVM, TMI, FMI, REV, CAGB	108
Roncella, 2013 [140]	Italy	101	MI, PCI	55.0	78.2%	UC	PM	TMI, REV	12
Saffi, 2014 [141]	Brazil	80	CAD	58.5	68.8%	UC	NC, RFM, PE	TM, TMI, REV, CABG, PCI	12
Salminen, 2005 [142]	Finland	268	CAD	73.9	40.3%	UC	PE, ET	TM	16
Santaularia, 2017 [143]	Spain	86	MI, Angina	59.5	83.7%	RFM	RFM, PM, ET	TM, HOSP, CVHOSP	12
Schneider, 2012 [144]	USA	201	CAD	59.1	56.2%	PE	PM	TM, CVM, TMI, NFMI, REV, HOSP, CVHOSP	108
Schuler, 1992 [145]	Germany	113	Angina	53.5	100%	PE	NC, RFM, PM, PE, ET	TM, CVM, TMI, NFMI, REV, CABG, PCI	12
Sebregts, 2005 [146]	Netherlands	204	MI, CABG	55.4	77.9%	RFM, ET	RFM, PM, PE, ET	TM, REV, CABG, PCI, HOSP, CVHOSP	12
Shaw, 1981 [147]	USA	651	MI	51.7	100%	UC	ET	TM, CVM, TMI, FMI, NFMI, REV, CABG, HOSP	36
Singh, 1992 [148]	India	505	MI, Angina	50.5	72.3%	NC	NC, PE	TM, CVM, TMI, FMI, NFMI	12
Singh, 2002 [149]	India	1000	MI, Angina, CAD	48.5	89.7%	NC	NC, PE	TM, CVM, TMI, FMI, NFMI, REV	24
Sivarajan, 1982 [150] †	USA	258	MI	56.3	79.8%	UC	ET	TM, CVM, REV	6

Specchia, 1996 [151]	Italy	256	MI	52.9	91.4%	UC	ET	TM, CVM, REV, CABG, PCI	34.5
Stahle, 1999 [152]	Sweden	101	MI, Angina	71.0	80.2%	PE	PM, ET	REV, CABG, PCI, HOSP	12
Stern, 1983 [153] †	USA	106	MI	54.0	85.8%	UC	ET	TM, TMI, REV, CABG	36
Strandberg, 2006 [154]	Finland	400	MI, CAD	80.0	34.8%	UC	NC, RFM	TM, CVM, TMI	36
Todd, 1991 [155]	Scotland	40	Angina	52.0	100%	UC	ET	TM, TMI, NFMI	12
Toobert, 2000 [156]	USA	28	MI, CABG, PCI, Angina, CAD	63.5	NR	UC	NC, PM, ET	TM, CVM, TMI, FMI	24
Trzcieniecka-Green, 1996 [157]	UK	100	MI, CABG	60.2	87.0%	UC	PM	TMI, NFMI, REV, CABG	6
Turner, 2013 [158]	Australia	57	PCI, CABG, CAD	61.5	73.7%	PE	PM	TM	12
Vahedian, 2016 [159]	Iran	70	MI	61.4	65.7%	PE, ET	RFM, PM, PE, ET	TM	33
Vale, 2003 [160]	Australia	792	CABG, PCI, MI, Angina	58.4	77.0%	UC	NC, RFM, PE	TM	6
Van Dixhoorn, 1987 [161]	Netherlands	90	MI	55.1	93.3%	ET	PM, ET	TM, CVM, TMI, NFMI, REV, CABG, HOSP, CVHOSP	24
Vermeulen, 1983 [162]	Netherlands	98	MI	47.5	100%	UC	PM, ET	TM, CVM, TMI, FMI, NFMI	60
Vona, 2009 [163] †	Switzerland	611	CAD	NR	NR	UC	RFM, PE	HOSP	12
Vona, 2011 [164]	NR	604	MI	NR	NR	UC	ET	HOSP, CVHOSP	24
West, 2012 [165]	UK	1813	MI	64.4	73.5%	PE	PE, ET	TM, TMI, REV, CABG, PCI, HOSP, CVHOSP	108

Wheeler, 2003 [166]	USA	570	MI, Angina	72.0	NR	UC	PE	TM	18
Wilhelmse, 1975 [167]	USA	315	MI	50.6	88.9%	UC	ET	TM, CVM, TMI, FMI, NFMI	60
Xavier, 2016 [168]	India	806	MI, Angina	56.4	82.6%	UC	RFM, PE	TM	12
Yu, 2004 [169]	China	269	MI, PCI, Angina	64	75.8%	RFM, PE	RFM, PE, ET	TM, HOSP	24
Zetta, 2011 [170]	UK	233	CABG, PCI	65.4	63.9%	PE	RFM, PM, PE	REV	6
Zhu, 2014 [171] †	China	196	MI, Angina, PCI	63.8	73.0%	UC	PE, ET	HOSP	6

MI = myocardial infarction; CAD = Coronary arterial Disease; CABG = Coronary artery bypass grafting; PCI = Percutaneous coronary intervention; NR = not reported; NA = not applicable; USA = United States of America; UK = United Kingdom; UC = usual care; NC = Nutritional Counselling; RFM = Risk Factor Modification; PM = Psychosocial Management; PE = Patient Education; ET = Exercise Training. TM = total mortality. CVM = cardiovascular mortality. FMI = fatal myocardial infarction. NFMI = non-fatal myocardial infarction. REV = revascularization. HOSP = hospitalization. CVHOSP = cardiovascular hospitalization. † components in trials with 3 arms not shown.

Supplemental File 3—Core Components Evaluated in Study Arms of Included Trials by Endpoint

	Number of Arms with CR Component by Outcome									
**Component**	**Mort**	**CV Mort**	**MI**	**FMI**	**NFMI**	**REV**	**CABG**	**PCI**	**Hosp**	**CV Hosp**
UC	75	24	34	9	21	29	18	12	30	15
NC	4	4	3	2	3	2	0	1	0	0
PE	26	10	12	3	5	12	8	6	8	5
ET	21	13	13	4	11	14	11	4	8	5
PM	15	3	7	2	4	5	2	2	7	4
RFM	7	3	3	1	2	4	3	3	4	3
NC + PE	2	2	2	2	2	1	0	0	0	0
NC + ET	1	0	0	0	0	0	0	0	0	0
NC + PM	1	1	1	0	0	2	2	2	1	1
NC + RFM	2	2	2	0	0	0	1	1	0	0
PE + ET	8	1	3	1	0	1	1	1	3	2
PE + PM	9	3	4	0	6	7	3	1	4	4
PE + RFM	12	2	2	1	1	0	0	0	6	1
ET + PM	3	2	2	1	2	2	2	1	3	1
ET + RFM	3	0	1	0	0	2	2	2	1	1
PM + RFM	3	2	2	1	1	0	0	0	1	1
NC + PE + ET	4	2	2	1	0	2	1	0	1	1
NC + PE + PM	0	0	0	0	0	0	0	0	0	0
NC + PE + RFM	4	1	1	0	0	3	2	3	1	0
NC + ET + PM	1	1	1	1	0	1	1	1	0	0
NC + ET + RFM	1	0	0	0	0	0	0	0	0	0
NC +PM + RFM	1	0	1	0	0	0	1	0	0	0
PE + ET + PM	6	2	2	1	0	3	1	1	2	0
PE + ET + RFM	4	0	1	0	1	1	1	0	1	0
PE + PM + RFM	2	1	0	0	0	2	0	0	1	1
ET + PM + RFM	1	0	0	0	0	0	0	0	1	1
NC + PE + ET + PM	5	1	1	0	0	1	0	0	2	0
NC + PE + ET + RFM	4	2	2	1	0	1	1	1	1	1
NC + PE + PM + RFM	0	0	0	0	0	0	0	0	0	0
NC + ET + PM + RFM	2	0	2	0	1	1	2	1	0	0
PE + ET + PM + RFM	4	0	0	0	0	1	1	1	1	1
NC+ PE + ET + PM + RFM	8	3	3	1	2	3	3	2	4	1
Total	239	85	107	32	62	100	67	46	91	49

CR = cardiac rehabilitation. NC = nutritional counseling. RFM = risk factor modification. PM = psychosocial management. PE = patient education. ET = exercise training; UC = usual care. Mort = mortality. CV = cardiovascular. MI = myocardial infarction. FMI = fatal myocardial infarction. NFMI = non-fatal myocardial infarction. Rev = revascularization. CABG = coronary artery bypass surgery. PCI = percutaneous coronary intervention. Hosp = hospitalization.

Supplemental File 4—Graphical Display of Distribution for Each Core Component for Each Outcome (Main-Effects Model)

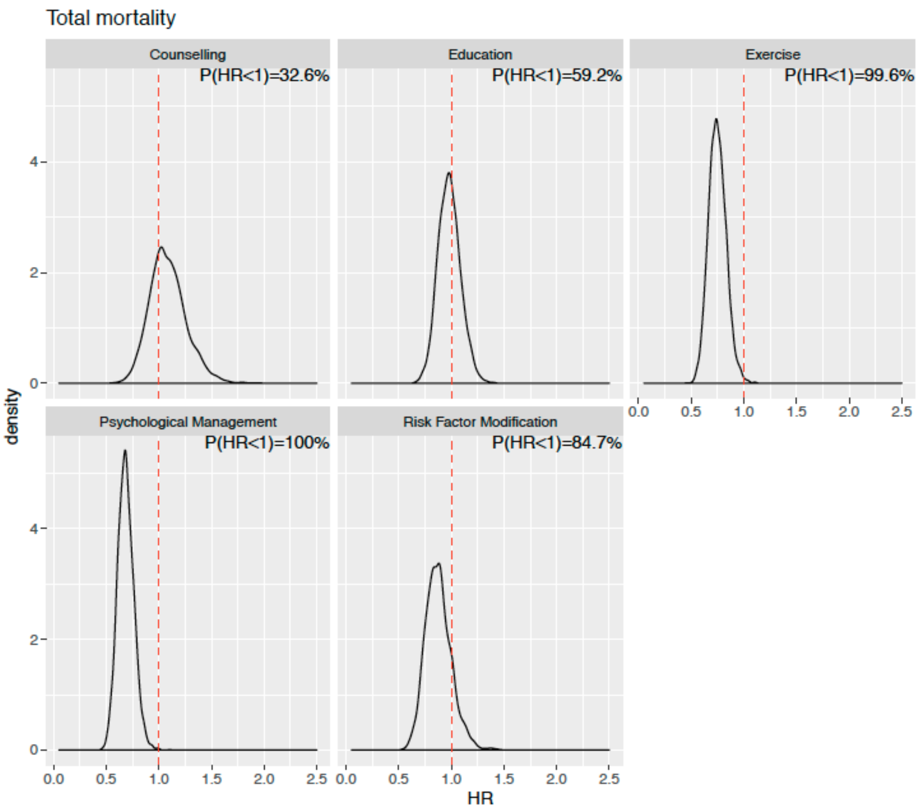

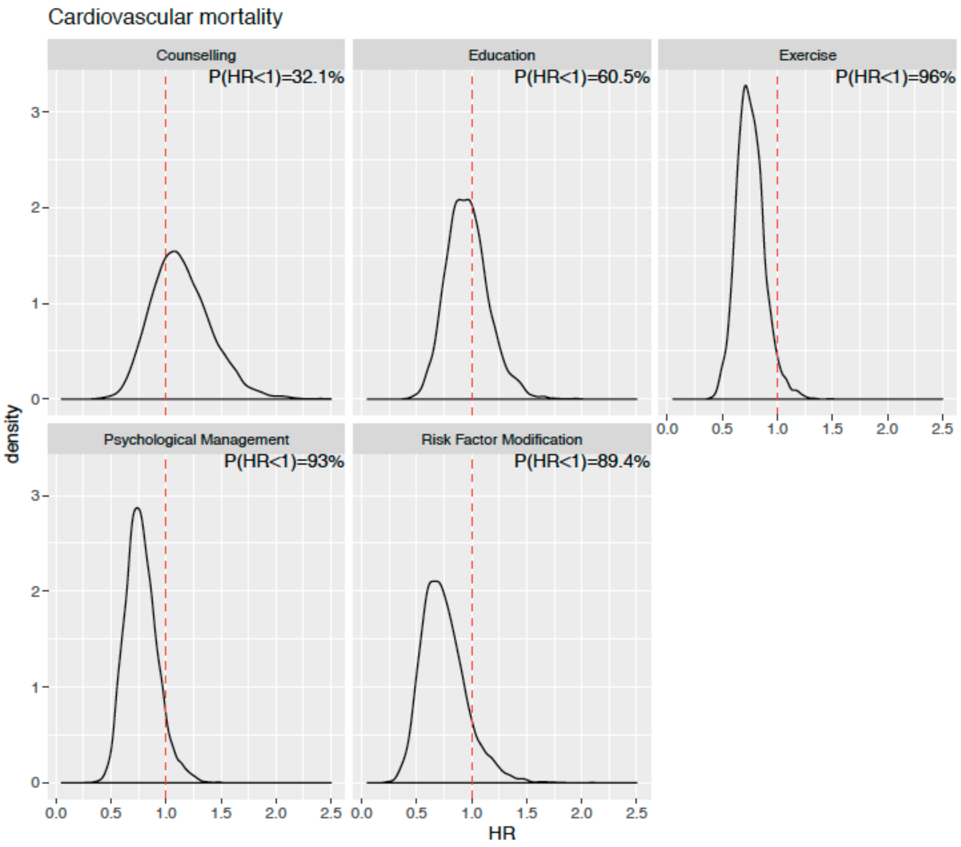

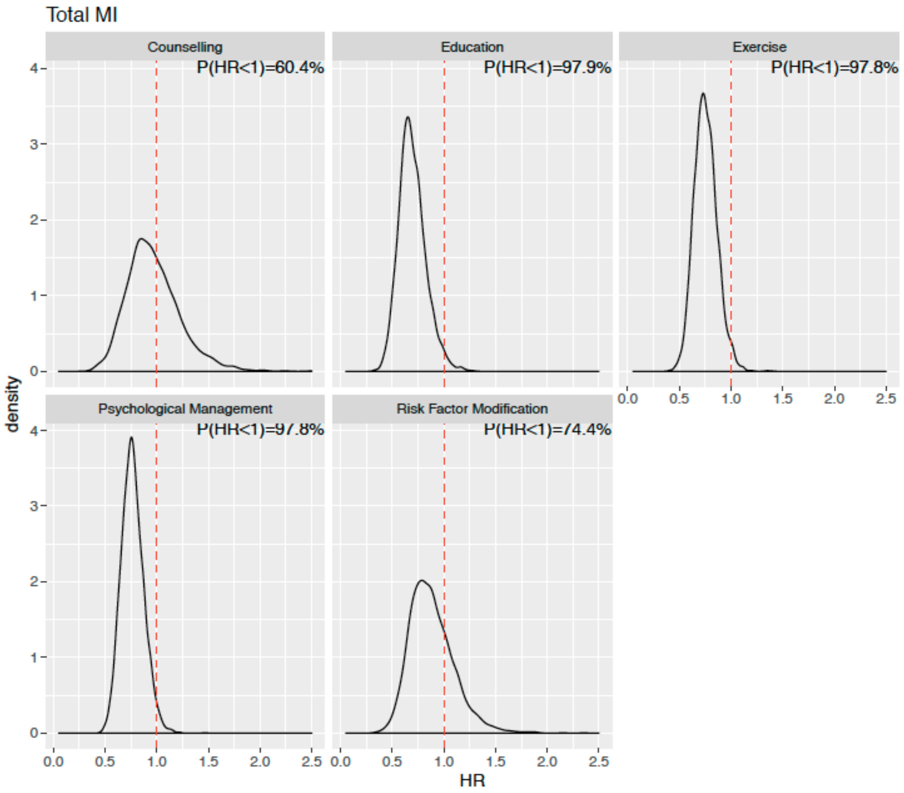
Total MI
Counselling
P(HR<1)=60.4%
Education
P(HR<1)=97.9%
Exercise
P(HR<1)=97.8%
Psychological Management
P(HR<1)=97.8%
Risk Factor Modification
P(HR<1)=74.4%
density
HR

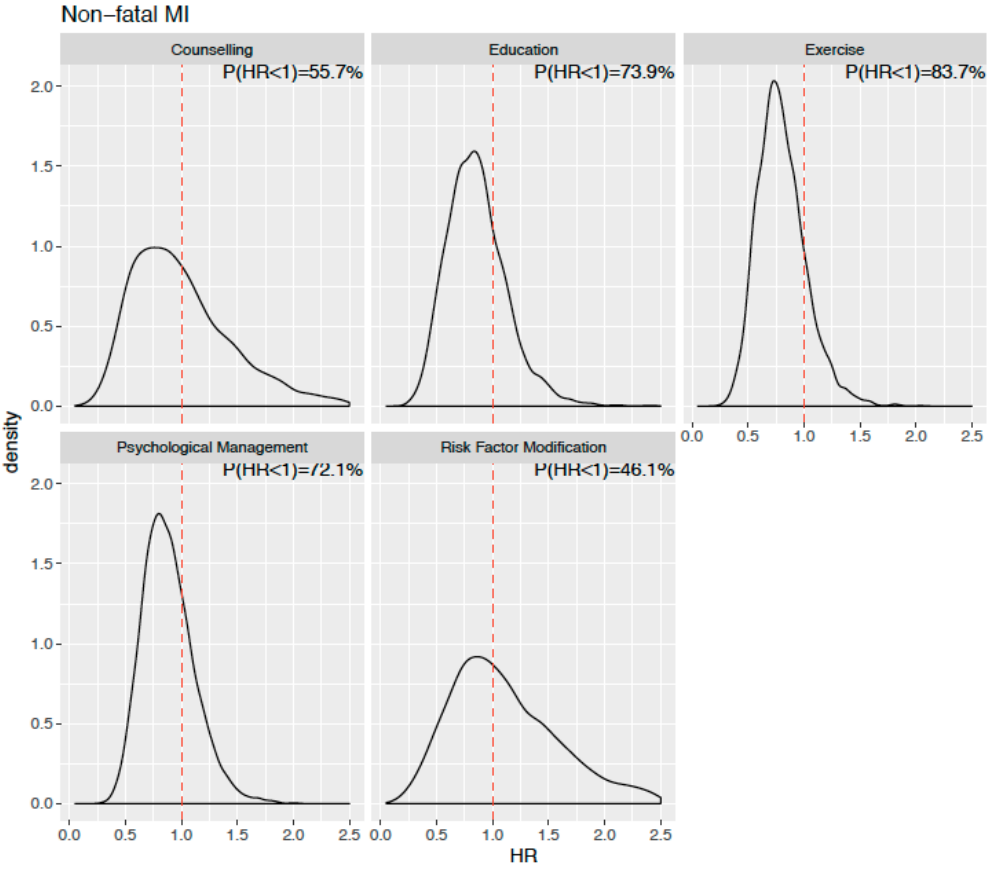
Non−fatal MI
Counselling
P(HR<1)=55.7%
Education
P(HR<1)=73.9%
Exercise
P(HR<1)=83.7%
Psychological Management
P(HR<1)=72.1%
Risk Factor Modification
P(HR<1)=46.1%
density
HR

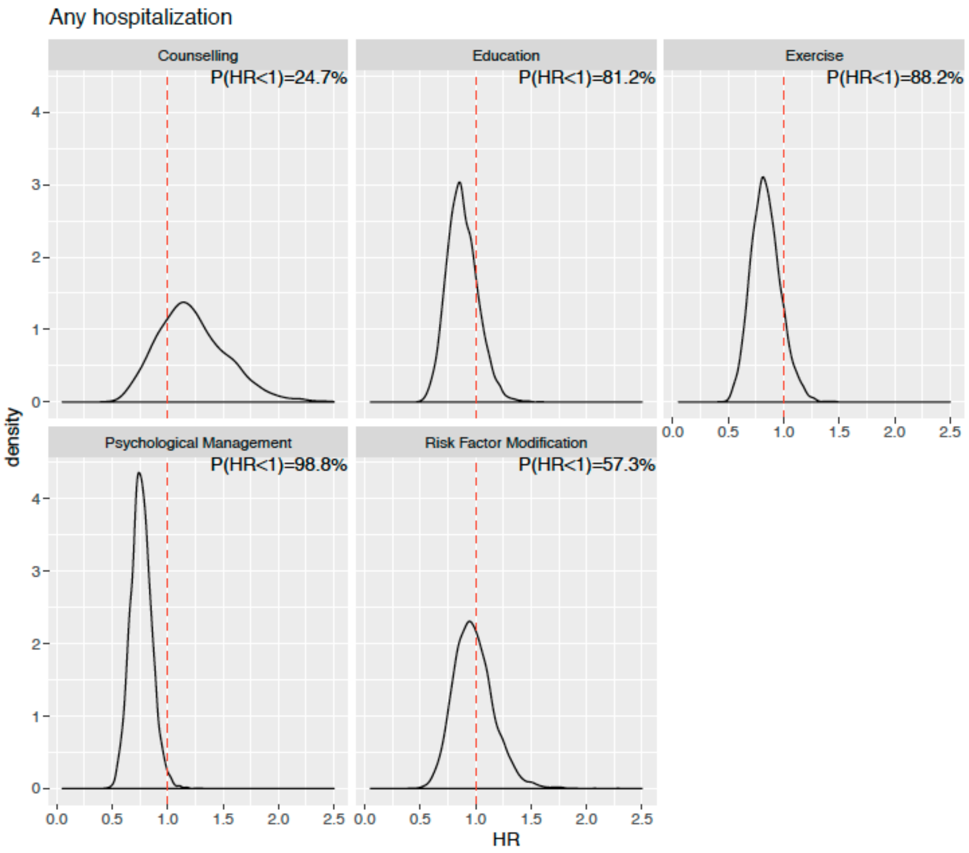

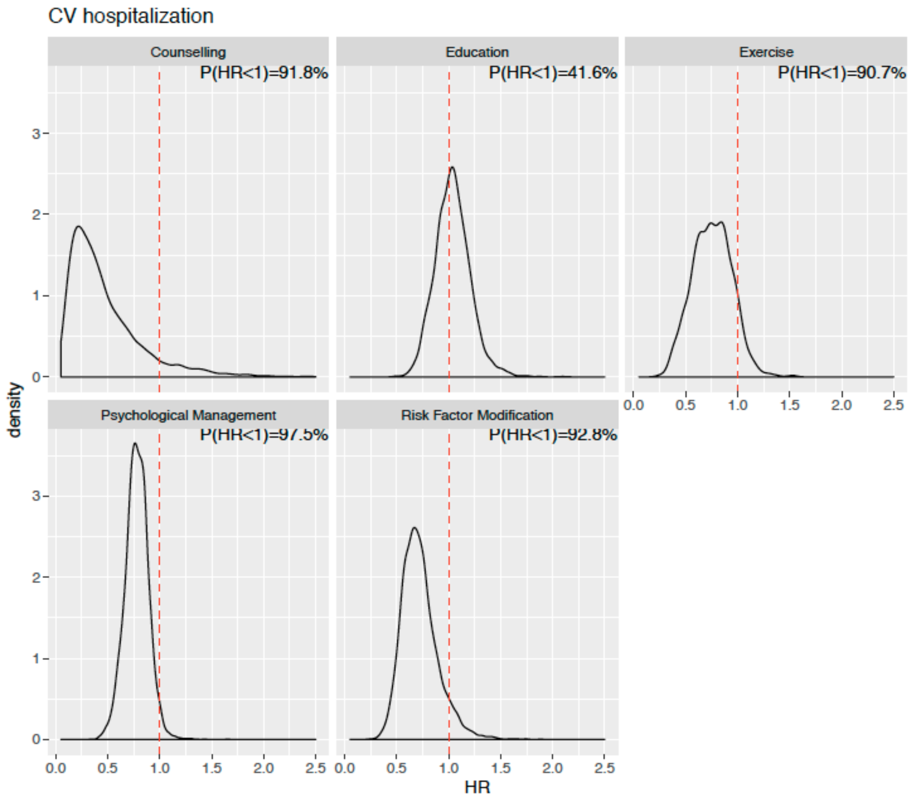

Note: counselling refers to nutrition counselling. CV, cardiovascular; MI, myocardial infarction.
